# Development of Two Distinct Dendritic-Like APCs in the Context of Splenic Stroma

**DOI:** 10.3389/fimmu.2013.00073

**Published:** 2013-03-20

**Authors:** Pravin Periasamy, Sawang Petvises, Helen C. O’Neill

**Affiliations:** ^1^Research School of Biology, Australian National UniversityCanberra, ACT, Australia

**Keywords:** stroma, hematopoiesis, hematopoietic stem cells, dendritic cells

## Abstract

Murine splenic stroma has been found to provide an *in vitro* niche for hematopoiesis of dendritic-like APC. Two distinct cell types have been characterized. The novel “L-DC” subset has cross-presenting capacity, leading to activation of CD8^+^ T cells, but not activating CD4^+^ T cells, which is consistent with their CD11c^lo^CD11b^hi^MHC-II^−^ phenotype. For L-DC, an equivalent tissue-specific APC has been found only in spleen. A second population of CD11c^hi^CD11b^lo^MHC-II^+^ cells resembling conventional dendritic cells (cDC) can activate both CD4 and CD8 T cells. Production of L-DC but not cDC-like cells is now shown to be dependent on contact between the L-DC progenitor and stroma such that the presence of a Transwell membrane can prevent L-DC development. Since L-DC can be produced continuously *in vitro* in stromal co-cultures overlaid with bone marrow (BM) progenitors, it was hypothesized that L-DC progenitors are self-renewing. The L-DC progenitor is shown here to be defined by the Flt3^−^c-kit^+^Lin^−^Sca-1^+^ (F^−^KLS) subset of adult BM which contains primitive HSC. Since the less primitive F^+^KLS HSC subset also contains L-DC progenitors, Flt3 does not appear to be a defining marker for this progenitor. Precursors of the cDC-like subset are found only within the F^+^KLS subset and seed production of a transient population of APC. All data identify differentiation of L-DC from HSC, and of cDC-like cells from DC precursors, which occurs independently of inflammatory signals and is dependent on a splenic stromal microenvironment.

## Introduction

A novel dendritic-like cell type has been described in spleen, namely “L-DC” (O’Neill et al., 2011; Tan et al., 2011). Studies to identify a progenitor of L-DC have revealed a subset of lineage (Lin)^−^c-kit^lo^ cells in adult and neonatal spleen reflecting hematopoietic stem/progenitor cells (HSPC) (Tan et al., 2010; Tan and O’Neill, 2012; Periasamy et al., 2013). L-DC progenitors also exist in bone marrow (BM) (Periasamy et al., 2009; Periasamy and O’Neill, 2013) and fetal liver (Hinton et al., 2011). When BM depleted of Lin^+^ cells is co-cultured above the 5G3 splenic stromal line, the phenotypically distinct CD11c^lo^CD11b^hi^MHC-II^−^CD8α^−^ subset of L-DC is produced continuously, along with a transient population of CD11c^hi^CD11b^lo^MHC-II^+^CD8α^−^ cells resembling conventional dendritic cell (cDC) (Periasamy et al., 2009; Periasamy and O’Neill, 2013). The latter have been shown to reflect a distinct APC type, which are not developmentally linked with L-DC (Periasamy and O’Neill, 2013). The latter cells are thought to arise from preformed DC precursors present in BM and spleen which have limited replicative capacity when cultured over 5G3 stroma. The L-DC subset is phenotypically distinct from known subsets of plasmacytoid (p) DC and CD8α^+^ and CD8α^−^ subsets of cDC described in spleen (Wu and Liu, 2007), which have also been shown to arise *in vitro* from Flt3L supplemented cultures of fractionated BM (Naik et al., 2005). Since L-DC production is sustained for long periods in splenic stromal co-cultures, the question arises as to whether the L-DC progenitor reflects a self-renewing stem cell. One explanation is that hematopoietic stem cells (HSC) are maintained *in vitro* in contact with 5G3 stroma, and undergo restricted differentiation with long-term (LT) production of L-DC. This would suggest maintenance of HSC *in vitro*, which was not readily achievable in the past. Here we have tested the hypothesis that HSC in BM can undergo differentiation to produce L-DC, and conclude that this happens but only when HSC maintain contact with splenic stroma. The 5G3 splenic stromal cell line has been employed as an *in vitro* niche, and its ability to support HSC maintenance and myelopoiesis tested by flow cytometric analysis of cells produced over time. HSC in murine BM are commonly identified as Lin^−^c-kit^+^Sca-1^+^ (KLS) cells (Spangrude et al., 1988) reflecting a heterogeneous subset (Kondo et al., 2003; Papathanasiou et al., 2009). Different HSC subsets can be distinguished as short-term (ST) or LT based on the extent of their potential to reconstitute an irradiated host (Weissman, 2000). The Flt3(F)^−^KLS subset of BM contains a majority of LT-HSC, and the F^+^KLS subset contains ST-HSC (Lai et al., 2005), although a minor CD34^+^ subset of F^−^KLS cells also has ST reconstitution capacity (Yang et al., 2005). Here BM-derived HSC, as the F^−^KLS and F^+^KLS subsets, have been compared for capacity to seed 5G3 co-cultures for L-DC production under different conditions. Since hematopoiesis involving BM-derived HSC can be induced in response to toll-like receptor (TLR) 2/4 stimulation by infectious agents (Kincade, 2006; Nagai et al., 2006), the role of inflammatory signaling in L-DC development was also investigated using knockout mouse strains.

## Materials and Methods

### Animals

Specific pathogen-free C57BL/6J (*H-2K^b^*), C57BL/6.*Rag1^−/−^*, C57BL/6.Tg(TcraTcrb)1100Mjb (OT-I), and C57BL/6.SJL/J.OT-II.CD45.1 (OT-II) mice aged 6–8 weeks were purchased from the John Curtin School of Medical Research (JCSMR: Canberra, ACT, Australia). C57BL/6.*MyD88^−/−^*, C57BL/6.*TRIF^−/−^*, and C57BL/6.*MyD88^−/−^TRIF^−/−^* mice were purchased from the Walter and Eliza Hall Institute (Melbourne, VIC, Australia). Mice were housed and handled according to protocols approved by the Animal Experimentation Ethics Committee at the Australian National University (Canberra, ACT, Australia). BM and spleen cells were dissociated by forcing tissue through a fine wire sieve, followed by lysis of red blood cells as described previously (Periasamy et al., 2009).

### Cell fractionation

Lin^−^ BM was prepared by depleting BM of hematopoietic lineage cells. Biotin-labeled antibodies specific for CD5, CD45R, CD11b, Gr-1 (Ly-6G/C), 7–4, and Ter-119 (Lineage Depletion kit, Miltenyi Biotec: North Ryde, NSW, Australia) along with added antibody specific to CD11c, were absorbed to cells according to manufacturer’s protocol. Following antibody binding, MACS® anti-biotin microbeads (Miltenyi Biotec) were added, cells transferred to a MACS® MS column (Miltenyi Biotec) which was placed in the permanent magnet of a SuperMACS® II Separator (Miltenyi Biotec). Cells binding the superparamagnetic anti-biotin microbeads are retained in the MACS® MS column (Miltenyi Biotec). Flow-through cells were collected after washing with buffer. An aliquot of the Lin^−^ cell population was tested by flow cytometry for the presence of Lin^+^ cells to determine the efficiency of depletion.

T cells were purified from spleen by depletion of macrophages, B cells, and MHC-II^+^ APC using specific antibodies and anti-Ig Dynabeads® (Invitrogen Dynal: AS, Oslo, Norway) as described previously (Tan et al., 2010). Antibodies were specific for CD11b (clone M1/70), B220 (clone RA3-6B3), and IA^b/k^ (clone TIB120) (eBiosciences). For depletion of CD4^+^ or CD8^+^ T cells, either anti-CD4 (GK1.5) or anti-CD8 (53-6.7) was included in the antibody cocktail (eBiosciences: San Diego, CA, USA). Fractionated T cells were labeled with carboxyfluorescein diacetate succinimidyl ester (CFSE) for flow cytometric analysis of their proliferation as described previously (Tan et al., 2010). CFSE (Molecular Probes: Eugene, OR, USA) was added to cells to a final concentration of 10 μg/ml, samples vortexed immediately, and then incubated at room temperature for 5 min. Cells were washed twice before use.

Splenic CD11c^+^ DC were freshly isolated as control APC using anti-CD11c magnetic MACS® microbeads (Miltenyi) as described previously (Tan et al., 2010). The cell suspension was run into MACS® MS column, and the column washed to deplete unbound cells. After the final wash, the column was removed from the SuperMACS® (Miltenyi) magnet and placed over a fresh tube for elution of CD11c^+^ labeled cells.

### Cell culture

The cloned 5G3 stromal line which supports *in vitro* hematopoiesis, and conditions for culture have been described previously (Despars and O’Neill, 2006; Periasamy et al., 2009). 5G3 was maintained by scraping attached cells for passage into a new flask. In order to maintain the stability of this cloned line, frozen stocks were established and cell cultures discarded after five passages. In controlled co-culture experiments, stromal cells were dissociated and harvested using 0.25% trypsin-EDTA treatment before plating a given number of cells.

### Flow cytometry and cell sorting

Fluorochrome-labeled antibodies specific for cell surface markers were purchased from either Biolegend (San Gabriel, CA, USA) or eBiosciences; c-kit (CD117), Sca-1 (D7), Flt3 (A2F10), CD69 (H12F3), CD11b (M1/70), CD11c (N418), I-A^b^ (MHC-II) (AF6-120.1), CD115 (AF598), Sirpα (P84), TCR-Vα2 (B20.1), and Thy1.2 (30-H12). These were used at minimal saturating concentration in multi-color staining experiments according to previously described methods for staining and washing of labeled cells (Periasamy et al., 2009; Tan et al., 2010). “Fc block” specific for FcγII/IIIR (CD32/CD16) (eBiosciences) was used to block non-specific binding of antibody. In all experiments, propidium iodide (PI) at 1 μgm/ml was added to cells for flow cytometric discrimination of dead cells. For multi-color staining, multiple primary antibodies were added together in the first staining step. The specificity of antibody binding was monitored through use of isotype control antibodies.

Flow cytometry was performed on an LSRII FACS machine (Becton Dickinson: Franklin Lakes, NJ, USA). FACSDIVA software (Becton Dickinson) was used to set voltage parameters and event counts while running samples. For multi-color analysis, single color compensation controls were used to set compensation on the machine. FlowJo software (FlowJo: Ashland, OR, USA) was used to analyze data. Commonly, cell debris was gated out using a forward scatter (FSC) threshold of 100. Cells were further gated on the basis of side scatter (SSC) and absence of PI staining to detect “live PI^−^” cells. Post-acquisition gating was used to obtain information on cell subsets, and staining with isotype controls was used to set gates to distinguish specific antibody staining.

Cell sorting was used to isolate hematopoietic cell subsets based on expression of Sca-1, c-kit, and Flt3. BM cells were stained with antibodies and the Lineage Depletion cocktail of biotinylated antibodies followed by a secondary fluorochrome-conjugated streptavidin conjugate (Miltenyi Biotec) for exclusion of mature hematopoietic cells. Sorting was performed on a BD FACSAria™ II cell sorter (Becton Dickinson).

Endocytosis was measured flow cytometrically to assess capacity of cells to take up antigen by addition of 100 μg/ml OVA-FITC (Molecular Probes) in a total volume of 100 μl sDMEM. Cells were incubated at 37°C for 45 min before endocytosis was halted by addition of 100 μl chilled PBS/0.1% NaN_3_. Cells were washed three times before analysis by flow cytometry.

### Establishment of co-cultures

The capacity of 5G3 to support hematopoiesis was assessed by overlay of Lin^−^ BM or HSC sorted from BM above stromal monolayers followed by co-culture for several weeks. This procedure has been described previously (Periasamy et al., 2009; Tan et al., 2010). Stromal cell lines were grown to 80–90% confluency, and Lin^−^ BM or HSC were plated at 1–5 × 10^4^ cells/ml above stromal monolayers. At 7-day intervals, non-adherent cells were collected by gently shaking the flask, with removal and replacement of supernatant.

### T cell activation assays

The antigen processing capacity of co-culture produced APC was measured by the ability of antigen-pulsed cells to induce proliferation of CD8^+^ T cells purified from spleens of OT-I TCR-Transgenic (Tg) mice specific for OVA_257-264_/H-2K^b^. The ability of isolated co-culture produced APC subsets to present antigen to CD4^+^ T cells similarly was measured using OT-II TCR-Tg mice specific for OVA_323-339_/H-2IA^b^. APC were pulsed with 10 μg/ml of OVA or control antigen HEL overnight (8–12 h), then washed twice by centrifugation. Some cultures were given lipopolysaccharide (LPS) as a potential activator (10 μg/ml) for 8–12 h.

CFSE-labeled T cells were cultured at 2 × 10^5^ cells per well in a 96-well plate together with graded numbers of APC, or alone as a control. Cells were plated in a total volume of 200 μl sDMEM, and plates incubated at 37°C for either 12 h or 4 days. T cells were identified by labeling for CD11c, TCR-Vα2, Thy1.2, and either CD4 or CD8. At 24 h, cells were also stained for CD69 expression to determine activation. At 4 days, CFSE staining was assessed as an indicator of cell proliferation. Cell division was assessed flow cytometrically in terms of reduction in CFSE level as T cells divide.

### Microscopy

A DM IRE2 inverted research microscope (Leica) equipped with DFC digital camera (Leica) was used to obtain phase contrast photomicrographs. Images were processed using Leica IM software v4.0.

### Statistical analysis

Student’s two-tailed *t*-test was used to test significance (*p* ≤ 0.05).

## Results

### Stromal cell contact influences myelopoiesis in co-cultures

**Figure 1:**
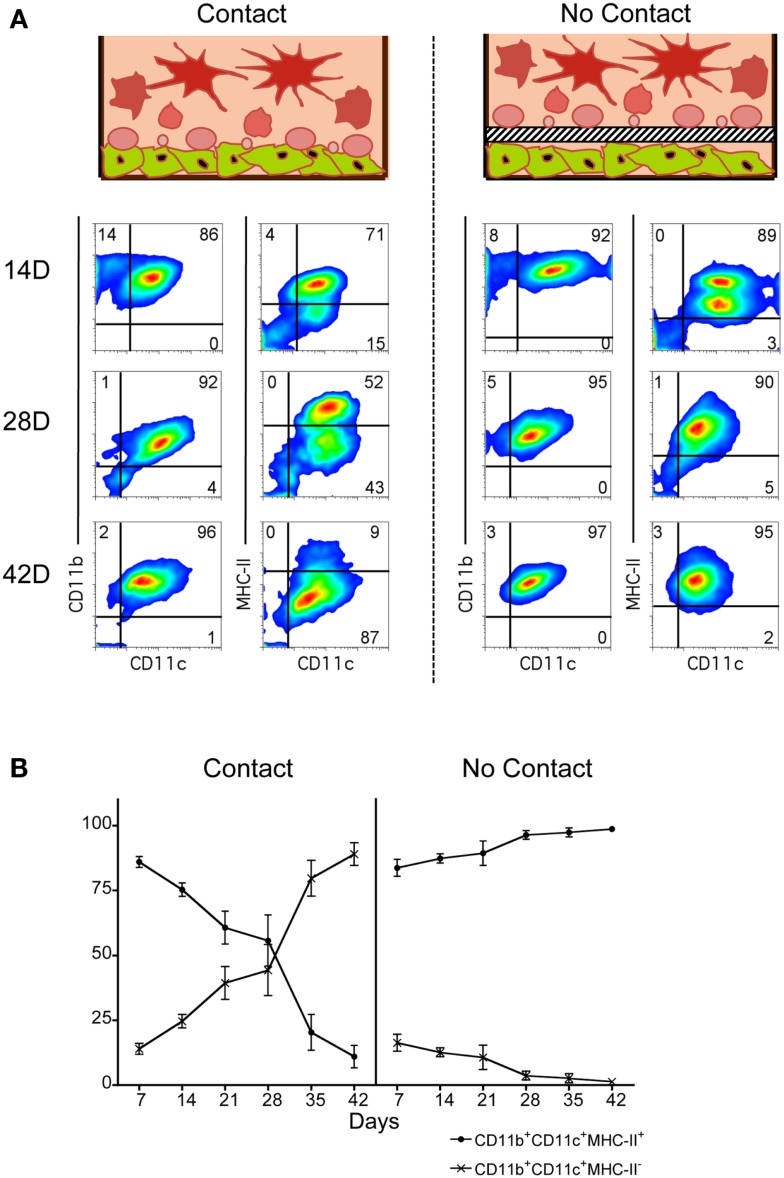
**Importance of stromal cell contact for L-DC development**. Co-cultures were established in three replicate experiments by overlay of Lin^−^ BM over near-confluent 5G3 stroma with or without a Transwell membrane for a period of up to 42 days. Non-adherent cells were collected at 7-day intervals and assessed in terms of cell surface phenotype. **(A)** Cells were stained with fluorochrome-conjugated antibodies specific for CD11c, CD11b, and MHC-II, or with isotype control antibodies. FSC and SSC analysis was used to gate large (FSC^hi^) cells (not shown) for subsequent multichannel analysis. Isotype controls were used to set gates indicating background antibody binding. Numbers shown in quadrants represent % positive cells. Data shown reflect one representative experiment out of three. **(B)** The relative proportion of MHC-II^−^ and MHC-II^+^ cells amongst CD11c^+^CD11b^+^ cells produced in co-cultures was estimated. Data represent mean ± SE (*n* = 3 replicate experiments).

Co-cultures of Lin^−^ BM over 5G3 stroma were established in the presence and absence of a Transwell membrane which prevents stromal contact with overlay cells. This barrier membrane enables assessment of the role of soluble factors in hematopoiesis in the absence of cell contact. Production of myeloid and dendritic cells was monitored by staining for CD11c, CD11b and MHC-II. Across 42 day co-cultures, cell production varied between co-cultures with and without Transwells. In the presence of a Transwell, almost all cells produced across 42 days were CD11c^+^CD11b^+^MHC-II^+^ cDC-like cells (Figure 1). These have been termed “Transwell” DC or “T-DC.” In co-cultures without a Transwell membrane, cell production across 14, 28, and 42 days involved an increasing proportion of CD11c^lo^CD11b^hi^MHC-II^−^ L-DC (Figure 1), with reducing numbers of CD11c^hi^CD11b^lo^MHC-II^+^ cDC-like cells as described previously (Periasamy et al., 2009). This result verifies the importance of progenitor contact with stroma for CD11c^+^CD11b^+^MHC-II^−^ L-DC production. A minor subset of CD11c^-^CD11b^+^MHC-II^−^ myeloid-like cells was detected early in co-cultures, suggesting that these cells developed from existing precursors in a contact-independent manner.

When overlay cells were in contact with stroma, an early population of CD11c^+^CD11b^+^MHC-II^+^ cDC-like cells was observed, representing ∼85% of all CD11c^+^CD11b^+^ cells at 7 days, but reducing to only ∼10% by 42 days (Figure 1B). In contrast, the CD11c^+^CD11b^+^MHC-II^−^ L-DC population increased from ∼15 to ∼87% over this time. The effect of Transwells was to reduce overall cell production, as well as specifically exclude production of L-DC. Lin^−^ BM co-cultures showed peak cell production at 28 days, and this was significantly greater (*p* < 0.01, Wilcoxon Rank Sum Test) than cell production in Transwell co-cultures (data not shown). These experiments emphasize the importance of stromal cell contact for L-DC production, and the role of soluble factors in the development of cDC-like cells (T-DC).

**Figure 2:**
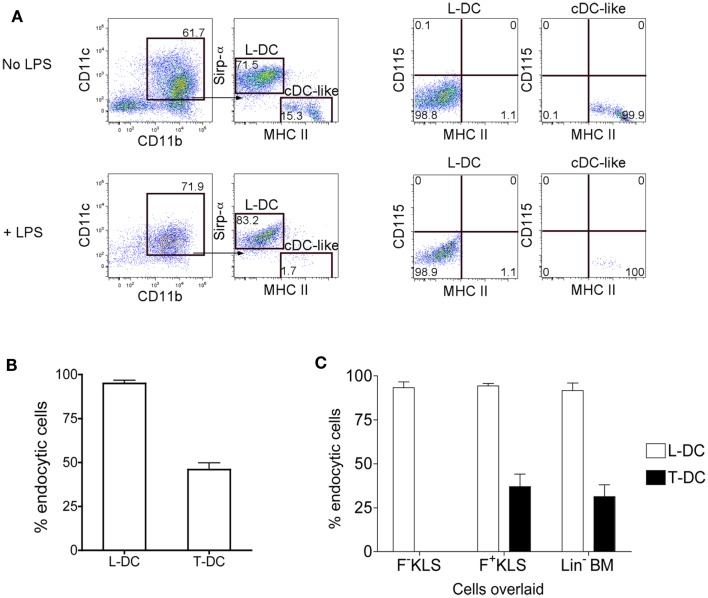
**Endocytic capacity of L-DC produced in co-cultures**. Co-cultures were established with Lin^−^ BM over 5G3 stroma as in Figure 1 with or without Transwells. **(A)** Non-adherent cells collected at 28 days were incubated for 24 h with and without LPS (10 μg/ml) and cells then stained with fluorochrome-conjugated CD11b, CD11c, MHC-II, Sirpα, and CD115 antibodies. Propidium iodide (PI; 1 μg/ml) was added to allow gating of PI^−^ live cells. BM cells served as a positive control to set gates for CD115 staining (data not shown). **(B)** Non-adherent cells collected at 28 days were also stained with antibody and sorted to isolate the predominant subsets of CD11b^+^CD11c^+^MHC-II^+^ cells (T-DC) produced in Transwell co-cultures (no contact), and CD11b^+^CD11c^+^MHC-II^−^ cells (L-DC) produced in normal co-cultures (contact). Sorted cells were compared for capacity to endocytose soluble antigen following incubation with FITC-conjugated ovalbumin (FITC-OVA; 2 mg/ml) for 45 min at 37°C, or at 4°C as control. Uptake of FITC-OVA was assessed flow cytometrically in terms of % endocytic cells. Data represent mean ± SE from three independent experiments. L-DC were significantly more endocytic than T-DC (*p* ≤ 0.05). **(C)** Co-cultures were established with HSC (F^−^KLS and F^+^KLS) and Lin^−^ BM, and sorted and analyzed as in **(B)**. L-DC were significantly more endocytic than T-DC in all three co-cultures (*p* ≤ 0.05).

Further evidence for production of 2 distinct cell types in co-cultures of Lin^−^ BM over 5G3 stroma was obtained when cells were stained for Sirpα, a marker present on some subsets of dendritic cells and on macrophages (Matozaki et al., 2009). The CD11c^+^CD11b^+^MHC-II^−^ subset of L-DC clearly expressed Sirpα^+^, while the CD11c^+^CD11b^+^MHC-II^+^ cDC-like cells did not (Figure 2A). Neither subset of cells expressed CD115 (M-CSFR), which distinguishes them from potential precursors of pDC and cDC in BM (Fancke et al., 2008). Following culture of isolated cells with LPS for 24 h, there was no cell activation evident by upregulation of MHC-II expression. However, the proportion of live cDC-like cells was reduced suggesting that they may have become activated and subsequently apoptotic following LPS treatment (Figure 2A).

### Co-cultures produce two subsets of functionally distinct APC

Lin^−^ BM co-cultures were established in the presence and absence of a Transwell membrane. Non-adherent cells were collected after 28 days of co-culture, and dominant subsets purified by sorting. L-DC were sorted as CD11c^+^CD11b^+^MHC-II^−^ cells from stromal contact co-cultures (no Transwell), and CD11c^+^CD11b^+^MHC-II^+^ cDC-like cells were sorted as T-DC from Transwell co-cultures. When these subsets were compared for endocytosis of FITC-OVA, as a measure of soluble antigen uptake capacity, L-DC were noticeably more endocytic than T-DC, with 96% of cells endocytic, compared with 45% for T-DC (Figure 2B).

**Figure 3:**
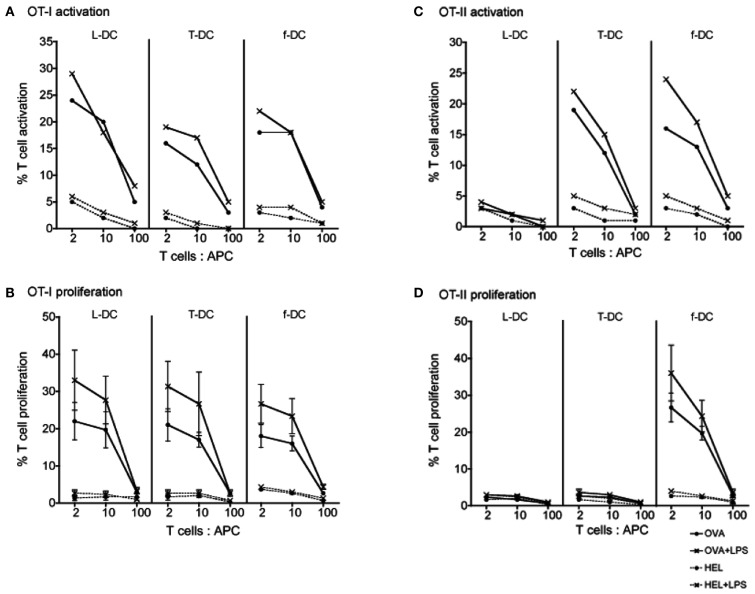
**Ability of L-DC and T-DC to activate T cells**. L-DC and T-DC produced in Lin^−^ BM co-cultures were prepared by sorting as described in Figure 2. They were compared for capacity to present soluble antigen OVA, or control antigen HEL, in the presence and absence of LPS to CD8^+^ T cells from OT-I TCR-Tg mice specific for OVA_257-264_/H-2K^b^ **(A,B)** and to CD4^+^ T cells from OT-II TCR-Tg mice specific for OVA_323-339_/H-2IA^b^ **(C,D)**. Freshly isolated splenic DC (f-DC) from C57BL/6J (*H-2^b^*) mice were used as an APC control. APC were pulsed for 12 h with OVA, HEL, OVA + LPS, or HEL + LPS. Diluting numbers of APC were co-cultured with different T cell: APC ratios and with CFSE-labeled CD8^+^ or CD4^+^ T cells purified from OT-I or OT-II spleens through depletion of B cells, myeloid cells, DC, and CD4^+^ or CD8^+^ T cells. **(A,C)** T cell activation was analyzed after 24 h in terms of % cells staining for CD69 in one of three replicate experiments. **(B,D)** T cell proliferation was measured after 4 days in terms of % cells showing a reduction in CFSE staining. T cells were gated as live (PI^−^) CD11c^−^Thy1.2^+^Vα2^+^ cells which were CD4^+^ or CD8^+^ using flow cytometry. Data represent mean ± SE of three replicate experiments. T cell only control (not shown) was <1%.

Sorted L-DC and T-DC were then compared along with freshly isolated CD11c^+^ spleen DC (f-DC) for ability to present exogenous antigen to CD8^+^ T cells purified from OT-I TCR-Tg mice. All three APC subsets were equally able to activate CD8^+^ OT-I T cells in an antigen (OVA)-specific manner. This was evident by upregulation of CD69 expression on gated CD8^+^Vα2^+^Thy1.2^+^ T cells at 24 h, both in the presence and absence of LPS (Figure 3A). After 4 days, all APC induced antigen-specific proliferation of CD8^+^ OT-I T cells measured in terms of percent cells showing reduction in CFSE, indicative of T cells which had divided at least once (Figure 3B). The addition of LPS caused a noticeable, although not significant, increase in CD8^+^ T cell proliferation at T cell: APC ratios of 2:1 and 10:1 for all APC tested (Figure 3B). This is consistent with the ability of LPS to upregulate MHC-I and CD80/86 expression on L-DC (Hinton and O’Neill, 2012), an effect also commonly described for cDC (Tan and O’Neill, 2005).

The ability of sorted L-DC and T-DC to present antigen to CD4^+^ T cells via the endocytic MHC-II pathway was tested using OT-II TCR-Tg mice. APC were pulsed with specific antigen OVA (and control antigen HEL) in the presence and absence of LPS. L-DC were unable to activate or stimulate proliferation of CD4^+^ T cells (Figure 3) consistent with their absence of MHC-II expression (Figure 2A). As expected, control f-DC showed antigen-specific, LPS-responsive activation of CD4^+^ T cells at 12 h, and T cell proliferation at 4 days (Figure 3). This was significantly enhanced by the presence of LPS at T cell: APC ratios of 2:1 and 10:1. While T-DC (cDC-like cells) were unable to stimulate CD4^+^ T cell proliferation after 4 days, although they did show strong antigen-specific, LPS-responsive activation of CD4^+^ T cells at 12 h. The restricted ability of T-DC, which are MHC-II^+^, to activate CD4^+^ T cells without subsequent T cell proliferation suggests inability of these cells to induce an immunogenic response. MHC-II expression on L-DC cannot be induced by LPS treatment (Hinton and O’Neill, 2012) in contrast to cDC where LPS causes upregulation of MHC-II (Tan and O’Neill, 2005).

### A purified HSC subset contains L-DC progenitors

**Figure 4:**
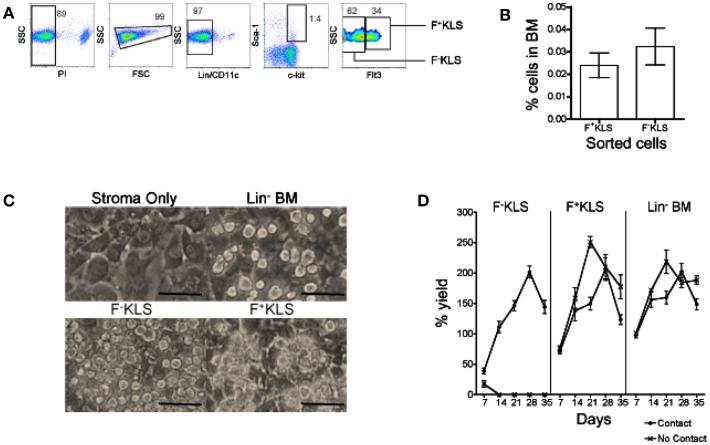
**Establishment of productive 5G3 co-cultures with HSC**. Lin^−^ BM was prepared using magnetic bead technology. Cells were then stained with a lineage antibody cocktail and antibodies specific for CD11c, c-kit, Sca-1, and Flt3 ahead of sorting to isolate HSC. **(A)** HSC were sorted as PI^−^Lin^−^CD11c^−^c-kit^+^Sca-1^+^Flt3^−^ cells (F^−^KLS), and PI^−^Lin^−^CD11c^−^c-kit^+^Sca-1^+^Flt3^+^ (F^+^KLS) cells. Gates were set using isotype controls, and numbers in gates represent % positive cells. **(B)** Recovery of HSC subsets from BM in three separate experiments. Data represent mean ± SE. **(C)** Sorted HSC subsets were co-cultured in contact with 5G3 stroma for up to 35 days, and photographed at 28 days under phase contrast microscopy (Magnification 200x, bar 100 μm). **(D)** % live cell recovery relative to total input cell number was estimated at 7-day intervals for co-cultures established with Transwells (no contact) and without Transwells (contact). Data represent mean ± SE for three replicate experiments.

Since stromal cell contact was required for L-DC production, the possibility that L-DC progenitors are closely related to HSC was considered. An HSC subset was prepared by sorting a Lin^−^ subset of BM following staining for lineage markers CD5, CD11b, B220, Ly-6G/C, 7–4, and Ter-119, and the DC marker CD11c. Cells were also stained for the hematopoietic progenitor markers c-kit, Sca-1, and Flt3 to gate two HSC subsets: Lin^−^CD11c^−^c-kit^+^Sca-1^+^Flt3^−^ (F-KLS) and Lin^−^CD11c^−^c-kit^+^Sca-1^+^Flt3^+^ (F^+^KLS) (Figure 4A). Sorted F^−^KLS represented ∼0.035% and sorted F^+^KLS ∼0.025% of all BM cells (Figure 4B). These two HSC subsets together with Lin^−^ BM as a positive control, were then tested for capacity to differentiate above 5G3 stroma in the presence and absence of a Transwell membrane. Three replicate experiments were established, and in each experiment, cells recovered from six flasks were pooled for analysis.

Figure 4C depicts the development of non-adherent cells in contact with the 5G3 stromal layer at 28 days in co-cultures established with Lin^−^ BM, and the F^−^KLS and F^+^KLS BM subsets. Stroma only control cultures showed no foci of developing cells. 5G3 stroma supported hematopoiesis in all co-cultures studied for 35 days. The cell yield relative to input cell number was calculated for co-cultures established with and without stromal cell contact. This reached a peak of ∼200% at 28 days for each of the F^−^KLS, F^+^KLS, and Lin^−^ BM co-cultures established with stromal cell contact (Figure 4D). Both the F^+^KLS and Lin^−^ BM subsets produced progeny cells over 35 days in both “contact” and “no contact” co-cultures. Co-cultures established with the most primitive F^−^KLS HSC were only productive in contact with stroma and not in the presence of a Transwell membrane.

**Figure 5:**
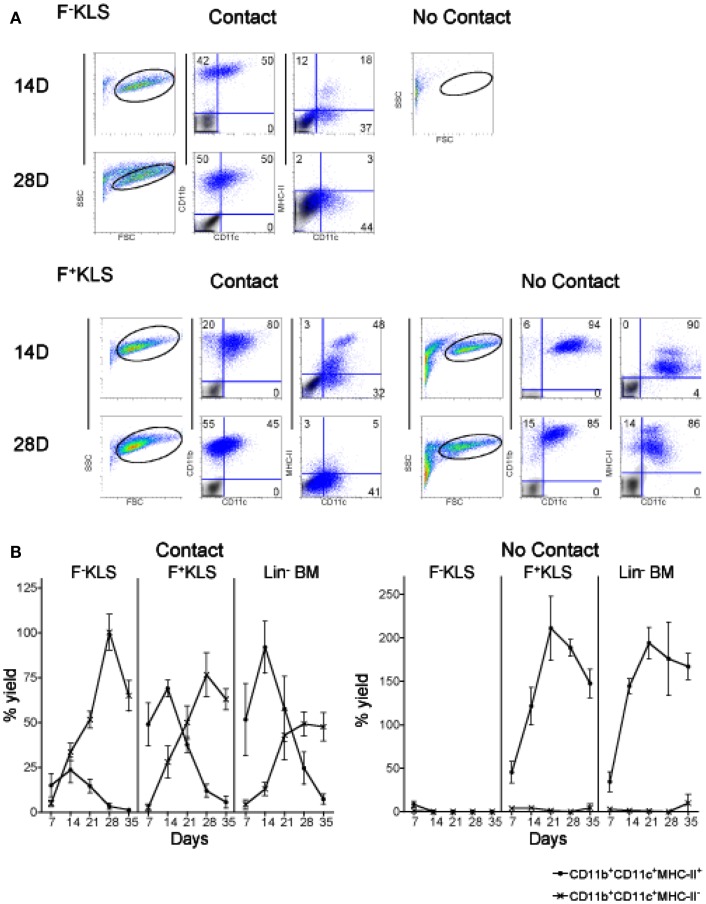
**HSC development in co-cultures**. Co-cultures were established by overlay of F^−^KLS or F^+^KLS subsets of HSC (sorted as in Figure 4) over near-confluent 5G3 stroma, with or without a Transwell membrane for a period of 35 days. Non-adherent cells collected at 7-day intervals were assessed in terms of cell yield and cell surface phenotype. **(A)** Cells were stained with fluorochrome-conjugated antibodies specific for CD11c, CD11b, and MHC-II, or with isotype control antibodies. FSC and SSC analysis was used to gate large cells for subsequent analysis. Isotype control staining, shown as a gray density plot overlay, was used to set gates indicating background binding. Numbers shown in quadrants represent % positive cells. **(B)** The percent yield of live CD11c^+^CD11b^+^MHC-II^−^ (L-DC) and CD11c^+^CD11b^+^MHC-II^+^ (cDC-like) cells in co-cultures was estimated relative to input cell number. Three replicate experiments were assessed. Data represent mean ± SE (*n* = 3). By day 28, production of L-DC was significantly greater than cDC-like cells (*p* ≤ 0.05) for all productive cultures.

Co-cultures established with each of the F^−^KLS and F^+^KLS HSC subsets of BM in contact with stroma remained viable cell producers for up to 35 days. Flow cytometric analysis of progeny from both F^−^KLS and F^+^KLS co-cultures established with 5G3 contact displayed predominantly an L-DC phenotype as CD11c^+^CD11b^+^MHC-II^−^ cells after 28 days (Figure 5A). This suggested that both the sorted F^−^KLS and F^+^KLS subsets must contain L-DC progenitors. The L-DC population was gated strictly on the basis of isotype controls which at early time points divided a single population of CD11b^+^ cells with a mixed CD11c^−/+^ phenotype. While one could argue that this population should be analyzed as one, further characterization has been deferred until useful markers are identified.

**Figure 6:**
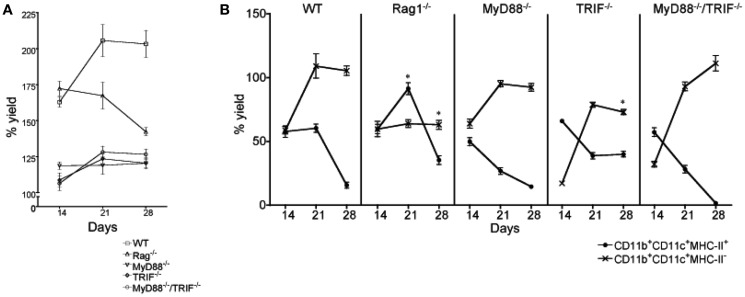
**Influence of inflammatory signals on L-DC hematopoiesis**. Mutant mice were compared with wild type (WT) mice to determine whether toll-like receptor (TLR) signaling or lymphoid cells are necessary to initiate L-DC hematopoiesis. Co-cultures were established over near-confluent 5G3 stroma for 28 days by overlay of Lin^−^ BM from individual mice: *MyD88*^−/−^ (*n* = 5), *TRIF*^−/−^ (*n* = 4), *MyD88*^−/−^*TRIF*^−/−^ (*n* = 4), *Rag1*^−/−^ (*n* = 2), and from WT C57BL/6J mice (*n* = 5). **(A)** Percent live cell recovery relative to input cell number was determined. Data represent mean ± SE. At 21 days, yield from all mutant co-cultures was significantly different from WT (*p* ≤ 0.05). **(B)** Non-adherent cells were collected at 14, 21, and 28 days and stained with fluorochrome-conjugated antibodies specific for CD11c, CD11b, and MHC-II to detect subsets of L-DC and cDC-like cells as described in Figure 5. The % yield of live CD11c^+^CD11b^+^MHC-II^−^ L-DC and CD11c^+^CD11b^+^MHC-II^+^ cDC-like cells in co-cultures from individual mice was estimated relative to input cell number using flow cytometric analysis. Data represent mean ± SE. Data points identified by * are significantly different (*p* < 0.01) from WT.

In stromal contact co-cultures, transient production of CD11b^+^CD11c^+^MHC-II^+^ cDC-like cells reached a peak at day 14 and then declined to be lost by 28–35 days (Figure 5B). CD11c^+^CD11b^+^MHC-II^+^ cDC-like cells constituted only a maximum of ∼25% of cells produced at 14 days in F^−^KLS co-cultures, compared with ∼70% in F^+^KLS co-cultures, and ∼85% in Lin^−^ BM co-cultures after 14 days (Figure 6B). A distinct MHC-II^hi^ cDC-like cell population was also observed in F^+^KLS co-cultures for up to 21 days (Figure 5B), and in Lin^−^ BM co-cultures for up to 35 days (Figure 5B). This population was evident in F^−^KLS co-cultures by 14 days but was gone by 28 days (Figure 6A). In addition to the outgrowth of cDC-like cells, an early transient CD11b^+^CD11c^−^MHC-II^−^ myeloid cell population was observed at 7 and 14 days, probably arising from preformed myeloid precursors (Figure 5A).

When contact between progenitors and stroma was prevented with a Transwell membrane, no hematopoiesis was observed for F^-^KLS co-cultures, and L-DC production which occurred in contact cultures, was inhibited (Figure 5). Production of only cDC-like cells and no L-DC was evident in F^+^KLS and Lin^−^BM co-cultures. Transwell cultures of F^+^KLS and Lin^−^BM were able to maintain production of cDC-like cells or “T-DC” for 35 days in the absence of stromal cell contact suggesting that soluble factors produced by stroma must drive the production of cDC-like cells from pre-existing precursors maintained within the F^+^KLS subset and Lin^−^BM. Within the T-DC population, two subpopulations were observed, differing in level of MHC-II expression. The MHC-II^hi^ subpopulation disappeared after 28 days in Lin^−^ BM co-cultures, but remained to 35 days in F^+^KLS co-cultures (Figure 5A).

L-DC derived from both purified F^−^KLS and F^+^KLS subsets of HSC were highly endocytic (>90% cells) (Figure 2C), as shown previously for L-DC derived in Lin^−^ BM co-cultures (Figure 2B). T-DC were distinct, however, and only 35–40% of T-DC derived from F^+^KLS and Lin^−^ BM Transwell co-cultures were able to endocytose FITC-OVA. L-DC showed significantly greater endocytic capacity than T-DC (*p* < 0.01).

### Hematopoiesis in 5G3 co-cultures is not driven by inflammatory signals

One consideration with respect to hematopoiesis occurring in co-cultures, is whether inflammatory events involving TLR signaling support the production of L-DC and/or cDC-like cells. A cascade of signaling reactions follows TLR binding by ligands, dependent on the adaptor proteins MyD88 and TRIF. All TLR except TLR3 require MyD88, and while TLR3 is dependent on TRIF, TLR4 can utilize either MyD88 or TRIF adaptor proteins (O’Neill and Bowie, 2007). Lin^−^ BM cells from *MyD88*^−/−^, *TRIF*^−/−^, and *MyD88*^−/−^*TRIF*^−/−^ mice were assessed in relation to wild type (WT) mice for capacity to undergo *in vitro* hematopoiesis in 5G3 co-cultures for L-DC production. *Rag*^−/−^ mice which lack lymphoid cells, were also tested to determine the importance of mature lymphoid cells, and any factors they produce, in the development of L-DC progenitors.

In all Lin^−^ BM co-cultures from mutant mice, there was constant development and maintenance of cells above 5G3 stroma. This was evident by total cell production assessed over 28 days of co-culture (Figure 6A), and by microscopy (not shown). However the total cell production in WT co-cultures was highest, with cell production in *Rag*^−/−^ co-cultures equal to WT at 14 days, but declining thereafter (Figure 6A). Each of the *MyD88*^−/−^, *TRIF*^−/−^, and *MyD88*^−/−^*TRIF*^−/−^ co-cultures showed significantly lower production of cells (*p* < 0.01) compared with WT mice across 14–28 days of co-culture, suggesting a lower frequency of progenitors, or lower ability of progenitors to undergo hematopoiesis. The relative frequency of L-DC to cDC-like cells produced was also assessed over 14–28 days to determine whether the relative frequency of progenitors for the two subsets varied between mutant and WT mice.

After cell production had stabilized after 28 days, WT, *MyD88*^−/−^, and *MyD88*^−/−^*TRIF*^−/−^ co-cultures produced only L-DC (Figure 6B). This suggested that hematopoiesis of L-DC did not depend on TLR signaling induced by pathogens or pathogen products contaminating co-cultures. The production of L-DC in *TRIF*^−/−^ co-cultures was significantly lower than in WT co-cultures at 28 days, suggesting that signaling through TRIF may contribute to hematopoiesis. The effect of *TRIF* was however partial, and since production in *MyD88*^−/−^*TRIF*^−/−^ co-cultures resembled WT, there appears to be a co-dependency between these two molecules.

All WT and mutant co-cultures showed an initial outgrowth of cDC-like cells that declined by 28 days (Figure 6B). Reduction in numbers of cDC-like cells occurred across 14–28 days coinciding with increased L-DC production, such that by 28 days the difference in the L-DC and cDC-like populations was large and significant (*p* ≤ 0.01) across all WT and mutant co-cultures (Figure 6B). *MyD88*^−/−^*TRIF*^−/−^, *MyD88*^−/−^, and WT co-cultures showed almost complete loss of the cDC-like population by 28 days (Figure 6B). At 21 days, the proportion of cDC-like cells in *MyD88*^−/−^, *TRIF*^−/−^, and *MyD88*^−/−^*TRIF*^−/−^ mice was significantly less than in WT co-cultures such that production of cDC-like cells could be to some extent dependent on inflammatory signaling events involving TLR.

A small distinct population of CD11b^+^CD11c^−^MHC-II^−^ myeloid cells was observed in WT (∼20%) and *TRIF*^−/−^ (∼15%) co-cultures at 14 and 21 days but was absent in *MyD88*^−/−^ and *MyD88*^−/−^*/TRIF*^−/−^ co-cultures (data not shown). Lin^−^ BM from *MyD88*^−/−^ and *MyD88*^−/−^*TRIF*^−/−^ mice may not contain preformed myeloid cell precursors seen previously in co-culture experiments in Figures 1 and 4. It is interesting to speculate that their development may be reliant on MyD88-dependent TLR signaling.

Lin^−^ BM isolated from *Rag1*^−/−^ mice cultured over 5G3 stroma gave a significantly lower yield of L-DC at days 21 and 28 compared with WT co-cultures (Figure 6B). L-DC development may therefore be influenced by lymphocytes, or their secreted factors. A significant increase in the yield of cDC-like cells after 21 days in *Rag1*^−/−^ versus WT co-cultures begs an explanation in terms of changes in precursor numbers within BM of *Rag1*^−/−^ mice.

## Discussion

The most significant finding to come from this work is the importance of 5G3 stromal cell contact in the survival, maintenance, and self-renewal of L-DC progenitors which can be isolated along with known HSC subsets of BM. With the use of Transwell co-cultures, it has been possible to distinguish the production of L-DC from those of the cDC-like cells (also termed here “T-DC”), only the latter developing in co-cultures when contact is restricted. The best explanation for the development of cDC-like cells under conditions of restricted contact is the preferential outgrowth of these cells under conditions which support their development, and clearly depends on contact with stroma, while cDC-like cell development occurs preferentially in the absence of L-DC development, supported by soluble factors. Development of cDC-like cells is also transient and ceases by ∼4 weeks, while production of L-DC can be maintained for up to 12 months (Periasamy et al., 2009; Hinton and O’Neill, 2011). L-DC are also shown here to be distinct from cDC-like cells by their expression of Sirpα, a marker common to macrophages and some DC subsets (Matozaki et al., 2009). They are also distinct by their inability to respond to LPS activation (Hinton and O’Neill, 2012). Indeed MHC-II expression is not upregulated upon LPS treatment, a result consistent with the inability of L-DC to activate CD4^+^ OT-II T cells in the presence and absence of LPS.

Since 5G3 stroma has been reported to produce M-CSF but not Flt3L or GM-CSF (Despars et al., 2004; O’Neill et al., 2004), these dendritic-like cells are distinct from cDC and pDC which develop *in vitro* from Flt3L-dependent precursors (Naik et al., 2005). Development of L-DC is also not dependent on GM-CSF, a factor that supports monocyte-derived (mo) DC development *in vitro* (Caux et al., 1992; Xu et al., 1995). A role for M-CSF in development of pDC and cDC from BM precursors has recently been reported (Fancke et al., 2008). However since neither L-DC or the cDC-like cells express CD115 (M-CSFR), they do not appear to reflect myeloid cells or progenitors which respond to M-CSF, although a role for M-CSF in development of L-DC and cDC-like cells from progenitors cannot yet be ruled out. Overall, these findings, along with phenotypic information, serve to distinguish L-DC and the cDC-like subset (T-DC) as distinct, and also distinct from known cDC, pDC, and moDC.

Another important finding is that the L-DC progenitor appears to be maintained in co-cultures and may be self-renewing. It is contained within both of the F^−^KLS and the F^+^KLS subsets of HSC sorted from BM. The F^+^KLS but not the F^−^KLS subset also contains precursors of cDC-like cells (or T-DC), which can differentiate for up to 35 days in co-cultures in the absence of stromal cell contact, apparently supported by soluble mediators. It is also significant that L-DC progenitors are unable to survive for more than 7 days in the absence of stromal cell contact. Since L-DC can be derived from the Flt3^−^ HSC subset in BM, they are further distinguished from cDC and pDC which derive from the Flt3^+^ common dendritic progenitor (CDP) (Onai et al., 2007; Liu et al., 2009), and from monocytes and cDC which derive from the Flt3^+^ myeloid dendritic progenitor (MDP) (Varol et al., 2007; Liu et al., 2009).

The best interpretation of results obtained here is that L-DC progenitors are present within both the F^−^KLS and F^+^KLS subsets of HSC, and that Flt3 is not a determining marker for this progenitor. A second explanation is that distinct but linked progenitors may exist, such that the progenitor contained within the F^−^KLS subset may differentiate upon stromal contact to give the L-DC progenitor contained within the F^+^KLS subset, or vice versa. Further marker analysis of these HSC subsets will be needed to delineate L-DC progenitors more fully. The F^+^KLS subset of BM HSC must contain both an L-DC progenitor and a precursor of cDC-like cells. One issue requiring an explanation is why cDC-like cells are lost over time in stromal contact co-cultures, where L-DC predominate over time, while in the absence of stromal contact, cDC-like cells prevail. Our prediction is that it could take ∼2 weeks for L-DC progenitors in the F^−^KLS and F^+^KLS subsets of HSC to establish sustained contact with stroma leading to self-renewal. Only once this occurs, may development become skewed toward L-DC production. On the basis of Flt3 expression, the precursor of cDC-like cells may be related to described subsets of CDP or MDP, which are both precursors of cDC-like cells (Liu et al., 2009).

It has also been established that hematopoiesis leading to L-DC development occurs independently of TLR signaling, and therefore independent of inflammatory events. This result supports our hypothesis for spleen as a site for hematopoiesis in the steady-state leading to the production of a tissue-specific APC, namely L-DC. However, TLR signaling through MyD88 appears to determine the outgrowth of a distinct CD11b^+^CD11c^−^MHC-II^−^ myeloid cell population in co-cultures involving the less pure population of Lin^−^ BM. This population is however transient, probably arising from a preformed myeloid precursor which develops in response to inflammation. The L-DC progenitor is quite distinct as a renewable progenitor, and its development is independent of TLR signaling. *MyD88*^−/−^ and *MyD88*^−/−^*TRIF*^−/−^ mice may therefore provide a better BM source of L-DC progenitors due to the absence of myelopoiesis induced by inflammation and TLR signaling. These could be utilized to benefit in future developmental studies.

The hallmark of DC is their unique ability to cross-present antigen to CD8^+^ T cells via the exogenous MHC-I pathway. Both L-DC and T-DC show capacity for cross-presentation of antigen to CD8^+^ T cells, activating them and inducing their proliferation. Further experiments are however underway to investigate the exact pathways for antigen processing by these distinct APCs. As cross-presenting cells, T-DC as MHC-II^+^ cells, resemble moDC or cDC (Belz et al., 2004; Dominguez and Ardavin, 2010). However, the absence of CD8α expression on T-DC (Periasamy and O’Neill, 2013) precludes their relationship with CD8α^+^ cDC (Naik et al., 2006), and their expression of CD11b precludes any similarity with the precursors of CD8α^+^ cDC, which are described as CD8α^−^CD11b^−^ cells (Bedoui et al., 2009).

In terms of antigen presentation to CD4^+^ T cells, L-DC and T-DC differ significantly. L-DC are unable to activate or induce proliferation of CD4^+^ T cells, while T-DC can activate CD4^+^ T cells, but not induce their proliferation. T cell activation and proliferation was not inducible with LPS treatment of T-DC, nor was it due to weak or absent MHC-II expression, since T-DC did induce antigen-specific activation of OT-II T cells. The T-DC subset described here is therefore also distinct as a cDC-like subset. One consideration is that these cells are an aberrant cell type induced by *in vitro* differentiation. Another is that they reflect a cDC-like or moDC-like cell which is immature, non-responsive to LPS, and non-immunogenic. Its classification as a tolerogenic or regulatory DC is possible, and is under further investigation. This would be consistent with the findings of others that regulatory DC can be derived by *in vitro* culture of progenitors from BM above stroma, although former studies did not use such pure populations of progenitors nor did they investigate the same T cell activation properties of regulatory DC produced (Svensson et al., 2004; Zhang et al., 2004; Tang et al., 2006). Notably, none of those former studies reported the production of L-DC.

Spleen is a known site for extramedullary hematopoiesis and a rescue niche for hematopoiesis when BM is compromised. HSC have been identified in resting spleen, albeit in lower numbers than in BM (Wolber et al., 2002; Dor et al., 2006; Tan and O’Neill, 2010). Here we confirm that spleen stroma can support myelopoiesis *in vitro* from HSC with continuous production of a novel dendritic-like “L-DC” reflecting tissue-specific hematopoiesis. Consistent with this finding is evidence that the *in vivo* equivalent of this novel cell type can also be identified amongst resting splenic myeloid and DC subsets in normal mice, and it is not present in other organs (Tan et al., 2011). This novel *in vivo* subset parallels in terms of phenotype and function, with cells generated *in vitro* both in LT spleen cultures (Quah et al., 2004) and in co-cultures of HSC over stroma as shown here.

## Authors Contribution

Pravin Periasamy: performance of experiments, analysis and assembly of data, and manuscript writing. Sawang Petvises: performance of experiments, analysis and assembly of data. Helen C. O’Neill: design of the project, interpretation and analysis of data, and manuscript writing.

## Conflict of Interest Statement

The authors declare that the research was conducted in the absence of any commercial or financial relationships that could be construed as a potential conflict of interest.

## References

[B1] Bedoui S. Prato S. Mintern J. Gebhardt T. Zhan Y. Lew A. M. (2009). Characterization of an immediate splenic precursor of CD8+ dendritic cells capable of inducing antiviral T cell responses. J. Immunol. 182, 4200–4207 10.4049/jimmunol.0802286 19299718

[B2] Belz G. T. Smith C. M. Kleinert L. Reading P. Brooks A. Shortman K. (2004). Distinct migrating and nonmigrating dendritic cell populations are involved in MHC class I-restricted antigen presentation after lung infection with virus. Proc. Natl. Acad. Sci. U.S.A. 101, 8670–8675 10.1073/pnas.0402644101 15163797 PMC423253

[B3] Caux C. Dezutter-Dambuyant C. Schmitt D. Banchereau J. (1992). GM-CSF and TNF-alpha cooperate in the generation of dendritic Langerhans cells. Nature 360, 258–261 10.1038/360258a0 1279441

[B4] Despars G. Ni K. Bouchard A. O’Neill T. J. O’Neill H. C. (2004). Molecular definition of an in vitro niche for dendritic cell development. Exp. Hematol. 32, 1182–1193 10.1016/j.exphem.2004.08.013 15588943

[B5] Despars G. O’Neill H. C. (2006). Splenic endothelial cell lines support development of dendritic cells from bone marrow. Stem Cells 24, 1496–1504 10.1634/stemcells.2005-0530 16769761

[B6] Dominguez P. M. Ardavin C. (2010). Differentiation and function of mouse monocyte-derived dendritic cells in steady state and inflammation. Immunol. Rev. 234, 90–104 10.1111/j.0105-2896.2009.00876.x 20193014

[B7] Dor F. J. Ramirez M. L. Parmar K. Altman E. L. Huang C. A. Down J. D. (2006). Primitive hematopoietic cell populations reside in the spleen: studies in the pig, baboon, and human. Exp. Hematol. 34, 1573–1582 10.1016/j.exphem.2006.06.016 17046577

[B8] Fancke B. Suter M. Hochrein H. O’Keeffe M. (2008). M-CSF: a novel plasmacytoid and conventional dendritic cell poietin. Blood 111, 150–159 10.1182/blood-2007-05-089292 17916748

[B9] Hinton R. A. O’Neill H. C. (2011). In vitro production of distinct dendritic-like antigen-presenting cells from self-renewing hematopoietic stem cells. J. Leukoc. Biol. 91, 341–346 10.1189/jlb.0611302 22075929

[B10] Hinton R. A. O’Neill H. C. (2012). “Extramedullary hematopoiesis leading to the production of a novel antigen-presenting cell type in murine spleen,” in Hematopoietic Stem Cells: New Research, eds Montgomery W. G. Burton H. I. (New York: Nova Science Publishers), 1–13 25157451

[B11] Hinton R. A. Papathanasiou P. O’Neill H. C. (2011). Distinct in vitro myelopoiesis is dependent on the self-renewal of hematopoietic progenitors. Scand. J. Immunol. 75, 168–175 10.1111/j.1365-3083.2011.02643.x 21958239

[B12] Kincade P. W. (2006). Supplying the demand for granulocytes. Nat. Immunol. 7, 701–702 10.1038/ni0706-701 16785885

[B13] Kondo M. Wagers A. J. Manz M. G. Prohaska S. S. Scherer D. C. Beilhack G. F. (2003). Biology of hematopoietic stem cells and progenitors: implications for clinical application. Annu. Rev. Immunol. 21, 759–806 10.1146/annurev.immunol.21.120601.141007 12615892

[B14] Lai A. Y. Lin S. M. Kondo M. (2005). Heterogeneity of Flt3-expressing multipotent progenitors in mouse bone marrow. J. Immunol. 175, 5016–5023 16210604 10.4049/jimmunol.175.8.5016

[B15] Liu K. Victora G. D. Schwickert T. A. Guermonprez P. Meredith M. M. Yao K. (2009). In vivo analysis of dendritic cell development and homeostasis. Science 324, 392–397 10.1126/science.1171243 19286519 PMC2803315

[B16] Matozaki T. Murata Y. Okazawa H. Ohnishi H. (2009). Functions and molecular mechanisms of the CD47-SIRPalpha signalling pathway. Trends Cell Biol. 19, 72–80 10.1016/j.tcb.2008.12.001 19144521

[B17] Nagai Y. Garrett K. P. Ohta S. Bahrun U. Kouro T. Akira S. (2006). Toll-like receptors on hematopoietic progenitor cells stimulate innate immune system replenishment. Immunity 24, 801–812 10.1016/j.immuni.2006.04.008 16782035 PMC1626529

[B18] Naik S. H. Metcalf D. Van Nieuwenhuijze A. Wicks I. Wu L. O’keeffe M. (2006). Intrasplenic steady-state dendritic cell precursors that are distinct from monocytes. Nat. Immunol. 7, 663–671 10.1038/nrg1954 16680143

[B19] Naik S. H. Proietto A. I. Wilson N. S. Dakic A. Schnorrer P. Fuchsberger M. (2005). Cutting edge: generation of splenic CD8+ and CD8 dendritic cell equivalents in Fms-like tyrosine kinase 3 ligand bone marrow cultures. J. Immunol. 174, 6592–6597 15905497 10.4049/jimmunol.174.11.6592

[B20] Onai N. Obata-Onai A. Schmid M. A. Ohteki T. Jarrossay D. Manz M. G. (2007). Identification of clonogenic common Flt3+M-CSFR+ plasmacytoid and conventional dendritic cell progenitors in mouse bone marrow. Nat. Immunol. 8, 1207–1216 10.1038/ni1518 17922016

[B21] O’Neill H. C. Griffiths K. L. Periasamy P. Hinton R. A. Hey Y. Y. Petvises S. (2011). Spleen as a site for hematopoiesis of a distinct APC type. Stem Cells Int. 2011, 954275 22190965 10.4061/2011/954275 PMC3236354

[B22] O’Neill H. C. Wilson H. L. Quah B. Abbey J. L. Despars G. Ni K. (2004). Dendritic cell development in long-term spleen stromal cultures. Stem Cells 22, 475–486 10.1634/stemcells.22-4-475 15277694

[B23] O’Neill L. A. J. Bowie A. G. (2007). The family of five: TIR-domain-containing adaptors in Toll-like receptor signalling. Nat. Rev. Immunol. 7, 353–364 10.1038/nri2079 17457343

[B24] Papathanasiou P. Attema J. L. Karsunky H. Jian X. Smale S. T. Weissman I. L. (2009). Evaluation of the long-term reconstituting subset of hematopoietic stem cells with CD150. Stem Cells 27, 2498–2508 10.1002/stem.170 19593793 PMC2783507

[B25] Periasamy P. O’Neill H. C. (2013). Stroma-dependent development of two dendritic-like cell types with distinct antigen presenting capability. Exp. Hematol. (in press). 10.1016/j.exphem.2012.11.003 23178375

[B26] Periasamy P. Tan J. K. H. Griffiths K. L. O’Neill H. C. (2009). Splenic stromal niches support hematopoiesis of dendritic-like cells from precursors in bone marrow and spleen. Exp. Hematol. 37, 1060–1071 10.1016/j.exphem.2009.06.001 19539692

[B27] Periasamy P. Tan J. K. H. O’Neill H. C. (2013). Novel spleen APCs derive from a Lin-ckitlo progenitor. J. Leukoc. Biol. 93, 63–39 10.1189/jlb.0512260 23099325

[B28] Quah B. Ni K. O’Neill H. C. (2004). In vitro hematopoiesis produces a distinct class of immature dendritic cells from spleen progenitors with limited T cell stimulation capacity. Int. Immunol. 16, 567–577 10.1093/intimm/dxh060 15039387

[B29] Spangrude G. J. Heimfeld S. Weissman I. L. (1988). Purification and characterization of mouse hematopoietic stem cells. Science 241, 58–62 10.1126/science.2898810 2898810

[B30] Svensson M. Maroof A. Ato M. Kaye P. M. (2004). Stromal cells direct local differentiation of regulatory dendritic cells. Immunity 21, 805–816 10.1016/j.immuni.2004.10.012 15589169

[B31] Tan J. K. O’Neill H. C. (2012). Myelopoiesis in spleen-producing distinct dendritic-like cells. J. Cell. Mol. Med. 16, 1924–1933 10.1111/j.1582-4934.2011.01490.x 22117595 PMC3822703

[B32] Tan J. K. Periasamy P. O’Neill H. C. (2010). Delineation of precursors in murine spleen that develop in contact with splenic endothelium to give novel dendritic-like cells. Blood 115, 3678–3685 10.1182/blood-2009-06-227108 20203267

[B33] Tan J. K. Quah B. J. Griffiths K. L. Periasamy P. Hey Y. Y. O’Neill H. C. (2011). Identification of a novel antigen cross-presenting cell type in spleen. J. Cell. Mol. Med. 15, 1189–1199 10.1111/j.1582-4934.2010.01089.x 20477902 PMC3822631

[B34] Tan J. K. H. O’Neill H. C. (2005). Maturation requirements for dendritic cells in T cell stimulation leading to tolerance versus immunity. J. Leukoc. Biol. 78, 319–324 10.1189/jlb.1104664 15809288

[B35] Tan J. K. H. O’Neill H. C. (2010). Haematopoietic stem cells in spleen have distinct differentiative potential for APCs. J. Cell. Mol. Med. 14, 2144–2150 10.1111/j.1582-4934.2009.00923.x 19799644 PMC3823005

[B36] Tang H. Guo Z. Zhang M. Wang J. Chen G. Cao X. (2006). Endothelial stroma programs hematopoietic stem cells to differentiate into regulatory dendritic cells through IL-10. Blood 108, 1189–1197 10.1182/blood-2006-01-007187 16627758

[B37] Varol C. Landsman L. Fogg D. K. Greenshtein L. Gildor B. Margalit R. (2007). Monocytes give rise to mucosal, but not splenic, conventional dendritic cells. J. Exp. Med. 204, 171–180 10.1084/jem.20061011 17190836 PMC2118434

[B38] Weissman I. L. (2000). Stem cells: units of development, units of regeneration, and units in evolution. Cell 100, 157–168 10.1016/S0092-8674(00)81692-X 10647940

[B39] Wolber F. M. Leonard E. Michael S. Orschell-Traycoff C. M. Yoder M. C. Srour E. F. (2002). Roles of spleen and liver in development of the murine hematopoietic system. Exp. Hematol. 30, 1010–1019 10.1016/S0301-472X(02)00881-0 12225792

[B40] Wu L. Liu Y. J. (2007). Development of dendritic-cell lineages. Immunity 26, 741–750 10.1016/j.immuni.2006.12.007 17582346

[B41] Xu H. Kramer M. Spengler H. P. Peters J. H. (1995). Dendritic cells differentiated from human monocytes through a combination of IL-4, GM-CSF and IFN-gamma exhibit phenotype and function of blood dendritic cells. Adv. Exp. Med. Biol. 378, 75–78 10.1007/978-1-4615-1971-3_15 8526149

[B42] Yang L. Bryder D. Adolfsson J. Nygren J. Mansson R. Sigvardsson M. (2005). Identification of Lin-Sca1+kit+CD34 +Flt3- short-term hematopoietic stem cells capable of rapidly reconstituting and rescuing myeloablated transplant recipients. Blood 105, 2717–2723 10.1182/blood-2004-06-2159 15572596

[B43] Zhang M. Tang H. Guo Z. An H. Zhu X. Song W. (2004). Splenic stroma drives mature dendritic cells to differentiate into regulatory dendritic cells. Nat. Immunol. 5, 1124–1133 10.1038/ni1130 15475957

